# ﻿An updated review of the genus *Toxorhina* Loew, 1850 (Diptera, Limoniidae) from Yunnan, China with a description of a new species

**DOI:** 10.3897/zookeys.1205.117807

**Published:** 2024-06-24

**Authors:** Hanhuiying Lv, Yuanyuan Xu, Shulin Liu, Linghui Li, Kejian Lin, Xiao Zhang

**Affiliations:** 1 Key Laboratory of Biohazard Monitoring and Green Prevention and Control in Artificial Grassland, Ministry of Agriculture and Rural Affairs, Institute of Grassland Research, Chinese Academy of Agricultural Sciences, Hohhot 010010, China Institute of Grassland Research, Chinese Academy of Agricultural Sciences Hohhot China; 2 Shandong Engineering Research Center for Environment-Friendly Agricultural Pest Management, College of Plant Health and Medicine, Qingdao Agricultural University, Qingdao 266109, China Qingdao Agricultural University Qingdao China; 3 Guangxi Key Laboratory of Agric-Environment and Agric-Products Safety, National Demonstration Center for Experimental Plant Science Education, Agricultural College, Guangxi University, Nanning 530004, China Guangxi University Nanning China

**Keywords:** *Ceratocheilus*, crane flies, Elephantomyiini, Limoniinae, male hypopygia, taxonomy

## Abstract

Seven species of the genus *Toxorhina* Loew, 1850 have been recorded from China, of which three are known to occur in Yunnan Province. Herein, all known species from Yunnan, China are reviewed with more detailed descriptions and illustrations of the male hypopygium. A species of Toxorhina belonging to the subgenus Ceratocheilus Wesché, 1910 from Yunnan, T. (C.) pianmica**sp. nov.**, is described and illustrated as new to science.

## ﻿Introduction

*Toxorhina* Loew, 1850 is a genus in the family Limoniidae (Diptera) with 152 extant species and subspecies, of which 79 species and two subspecies belong to the subgenus Ceratocheilus Wesché, 1910, 68 species belong to the subgenus Toxorhina and three species belong to the subgenus Eutoxorhina Alexander, 1934 (Oosterbroek 2023). Members of *Toxorhina* differ from most limoniid crane flies in their particularly elongate rostrum and are often recorded as feeding on flowers (e.g., Alexander and McAtee 1920; Alexander 1937; Lehnebach and Robertson 2004; Oosterbroek and Lukashevich 2021). The genera *Elephantomyia* Osten Sacken, 1860 and *Helius* Lepeletier & Serville, 1828 (in Latreille et al. 1828) have a similar elongate rostrum to *Toxorhina*, and these three genera, together with another limoniid genus without an elongate rostrum (i.e., *Protohelius* Alexander, 1928), form the tribe Elephantomyiini (Alexander 1920; Savchenko et al. 1992; Hynes 1997; Podenas and Gelhaus 2007; Kang et al. 2023).

Seven species of *Toxorhina* have been recorded from China, of which four (three new species and one newly-recorded species) were published in Zhang et al. (2015), in which all Chinese species were revised. After that, we had the opportunity to examine more specimens of Elephantomyiini collected from Yunnan Province, China, deposited in the Entomological Museum of China Agricultural University, Beijing, China (CAU). Yunnan is a part of the Yunnan-Guizhou Plateau and has a diverse environment and high species diversity; three species of *Toxorhina* are known to occur in the province (Zhang et al. 2015; Oosterbroek 2023). In this study, we obtain more specimens for all three known species of *Toxorhina* in Yunnan and provide more detailed descriptions and illustrations of the male hypopygium. In addition, a new species of *Toxorhina* from Yunnan, T. (C.) pianmica sp. nov., is also described and illustrated.

## ﻿Material and methods

**Figure 1.**
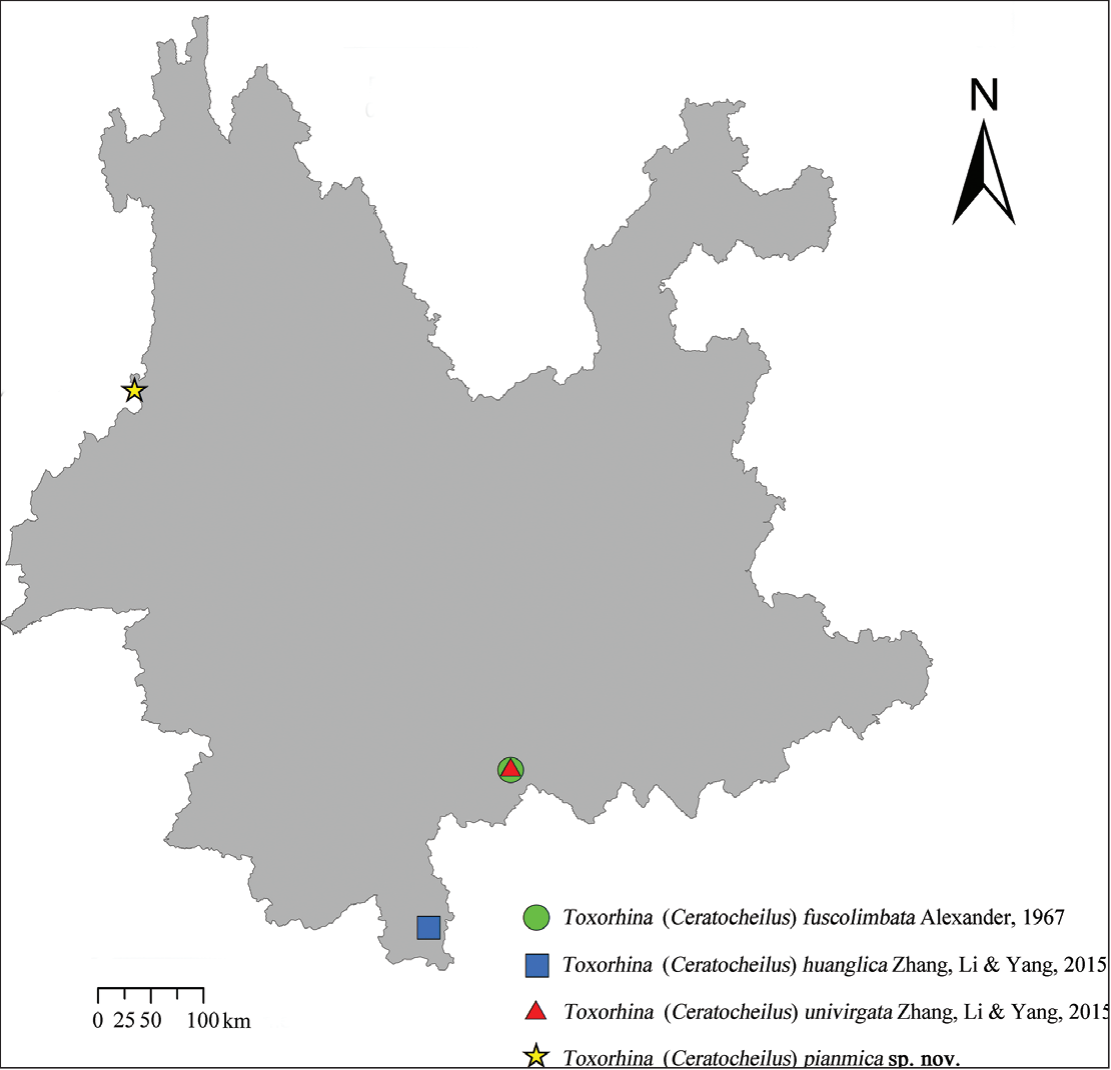
Collecting sites of *Toxorhina* species from Yunnan, China in this study. Toxorhina (Ceratocheilus) fuscolimbata and T. (C.) univirgata, Huanglianshan National Nature Reserve; T. (C.) huanglica, Xishuangbanna Tropical Rainforest Nature Reserve; T. (C.) pianmica, Pianma Town.

The specimens of this study were collected through light trapping at different locations in Yunnan, China (Fig. 1) and deposited in the CAU. Preparations of the male hypopygium were made by soaking dissected tip of abdomen in cold 10% hydroxide (NaOH) for 10 hours. Details of body coloration were examined in specimens immersed in 75% ethanol (C_2_H_5_OH). Prepared specimens were examined using a ZEISS Stemi 2000-C stereomicroscope. Photographs were captured using a Canon EOS 5D Mark IV digital camera through a Canon EF100 mm f/2.8L Macro IS USM lens, and the details of the male hypopygium were captured by that camera attached to a Phenix PH100-3B41L-IPL biomicroscope.

The morphological terminology mainly follows Cumming and Wood (2017) and de Jong (2017). The following abbreviations in figures are used: **aed** = aedeagus, **ea** = ejaculatory apodeme, **goncx** = gonocoxite, **gonst** = gonostylus, **i gonst** = inner gonostylus, **interb** = interbase, **o gonst** = outer gonostylus, **pm** = paramere, **st**= sternite, **tg** = tergite.

## ﻿Taxonomy

### ﻿Key to species of the genus *Toxorhina* from Yunnan

**Table:** 

1	Wing with black seams along cord and m-m (Zhang et al. 2015: fig. 3d). Gonocoxite with 2 gonostyli (Fig. 2A, B)	**Toxorhina (Ceratocheilus) fuscolimbata Alexander, 1967**
–	Wing without conspicuous seams along cord or m-m (Figs 4D, 6B, C; Zhang et al. 2015: figs 5d, 13d). Gonocoxite with 1 gonostylus (Figs 3A, B, 5A, B, 7A, B)	**2**
2	Pleuron without longitudinal stripe (Fig. 4A). Wing with cell d open by absence of m-m, R_4_ ending slightly before end of basal section of R_5_ (Fig. 4D). Gonocoxite dorsally with three brownish-black, stout preapical setae (Fig. 5A, F)	**Toxorhina (Ceratocheilus) pianmica sp. nov.**
–	Pleuron with one or two longitudinal stripes (Fig. 6A; Zhang et al. 2015: figs 3a, 5a, 13a). Wing with cell d closed, R_4_ ending beyond r-m (Fig. 6B, C; Zhang et al. 2015: figs 3d, 5d, 13d). Gonocoxite dorsally without stout preapical setae (Figs 2A, 3A, 7A)	**3**
3	Pleuron dark yellow with two black longitudinal stripes (Zhang et al. 2015: fig. 5a). Wing with R_4_ ending slightly beyond end of basal section of R_5_ (Zhang et al. 2015: fig. 5d). Gonocoxite dorsally with a blunt, basal lobe; lobe provided with numerous stout setae (Fig. 3A, G). Interbase elongated, rod-shaped, about 10 times as long as wide (Fig. 3A, B, C, E). Tip of aedeagus bifid with arms short (Fig. 3A, B, C, E, I)	**Toxorhina (Ceratocheilus) huanglica Zhang, Li & Yang, 2015**
–	Pleuron yellow with one dark brown longitudinal stripe (Fig. 6A; Zhang et al. 2015: fig. 13a). Wing with R_4_ ending beyond end of basal section of R_5_ by 1/3–2/5 of its own length (Fig. 6B, C; Zhang et al. 2015: fig. 13d). Gonocoxite dorsally without basal lobe (Fig. 7A). Interbase as a short and flattened plate, about 3 times as long as wide (Fig. 7A–D). Tip of aedeagus bifid, arms very long, filiform (Fig. 7A–D, H)	**Toxorhina (Ceratocheilus) univirgata Zhang, Li & Yang, 2015**

#### ﻿ Toxorhina (Ceratocheilus) fuscolimbata

Taxon classificationAnimaliaDipteraLimoniidae

Alexander, 1967

EBCD97A0-D349-5B77-A149-0EC6CC120400

Fig. 2

Toxorhina (Ceratocheilus) fuscolimbata Alexander, 1967 in Alexander 1967: 185. Type locality: India, Assam, Manipur, Hkayam Boum.
Toxorhina (Ceratocheilus) fuscolimbata in Zhang et al. 2015: 64.

##### Specimens examined.

China • 2 ♂♂; Yunnan Province, Lvchun County, Huanglianshan National Nature Reserve, Yakou Protection Station; 1931 m a.s.l.; 22.8956°N, 102.3008°E; 7 July 2016; Qilemoge leg.; light trap; CAU.

##### Diagnosis.

Prescutum and presutural scutum brownish-yellow with three broad and nearly confluent brownish-black longitudinal stripes. Pleuron yellow with two black longitudinal stripes. Wing with black seams along cord and m-m and paler seam over base of CuA. Cell d closed. R_4_ ending beyond end of basal section of R_5_ by half of its own length, m-cu beyond fork of M by 1/4–1/2 of its own length. Gonocoxite with 2 gonostyli. Gonocoxite dorsally without stout preapical setae. Interbase as a short and flattened plate. Tip of aedeagus bifid with arms short and divergent.

##### Description.

**Figure 2.**
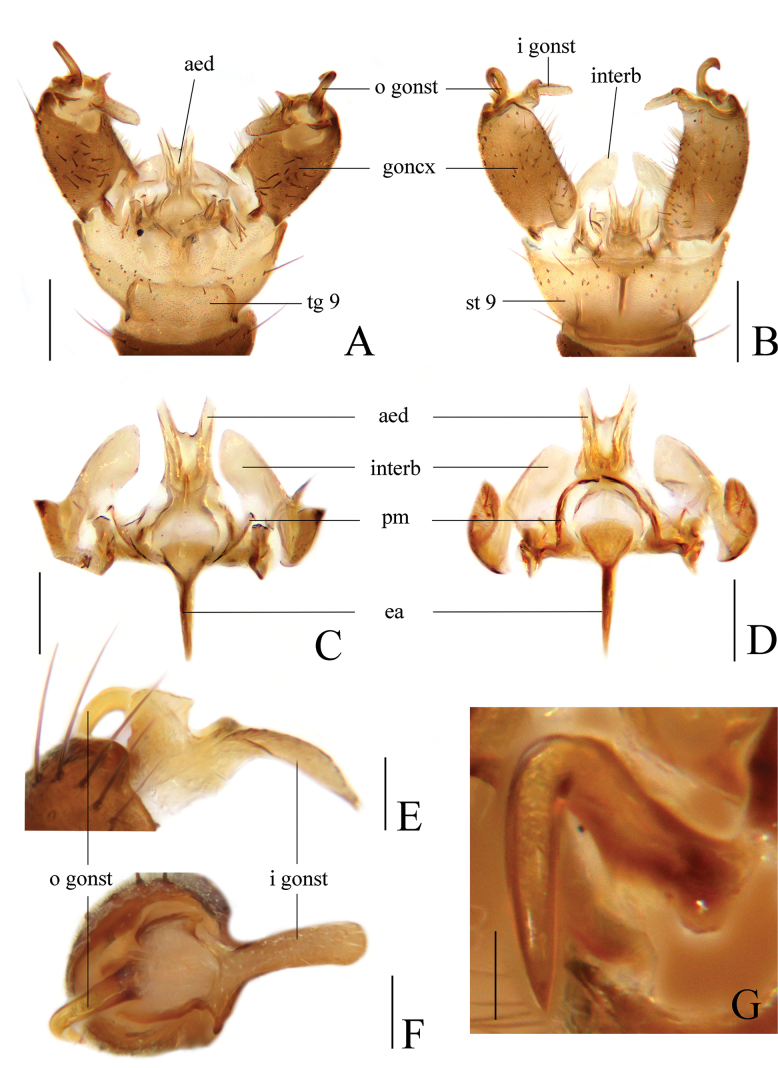
Toxorhina (Ceratocheilus) fuscolimbata**A** male hypopygium, dorsal view **B** male hypopygium, ventral view **C** aedeagal complex, dorsal view **D** aedeagal complex, ventral view **E** gonostyli, dorsal view **F** gonostyli, posterior view **G** outer gonostylus, posterior view. Scale bars: 0.2 mm (**A, B**); 0.1 mm (**C, D**); 0.05 mm (**E, F**); 0.02 mm (**G**).

**Male.** Hypopygium (Fig. 2). Tergite 9 brownish-yellow with dark brown setae, posterior margin with a broad and nearly rounded emargination (Fig. 2A). Gonocoxite dark brown, cylindrical, with dark brown setae; setae on dorsal side darker and stronger (Fig. 2A, B). Outer gonostylus dark brown, slender and rod-shaped, about 10 times as long as wide, strongly curved ventrally and inwards at middle; tip acute (Fig. 2A, B, E–G). Inner gonostylus brownish-yellow, about 4 times as long as wide, with rounded tip, basally provided with additional, ventral horn-like process (Fig. 2A, B, E, F). Interbase brownish-yellow, as a short and flattened plate, about 3 times as long as wide; tip blunt (Fig. 2A–D). Parameres dark brown, arched and medially fused, distally connecting to base of interbase (Fig. 2C, D). Ejaculatory apodeme dark brown, rod-shaped and straight, about 7 times as long as wide (Fig. 2C, D). Aedeagus brownish-yellow, stout at base, tip bifid with arms short and divergent (Fig. 2A–D).

##### Distribution.

China (Guangxi, Tibet, Yunnan), India (Oosterbroek 2023).

##### Behavior.

The species can be attracted by light.

##### Remarks.

Alexander (1967) first described this species based on one male and two female specimens collected in India, and later added figures of the wing and male hypopygium for this species (Alexander 1970). Zhang et al. (2015) reported this species as a new record from China and redescribed and illustrated it. A more detailed description and illustration of the male hypopygium of this species are provided in this study.

#### ﻿ Toxorhina (Ceratocheilus) huanglica

Taxon classificationAnimaliaDipteraLimoniidae

Zhang, Li & Yang, 2015

BD6B0F2A-C561-51FD-8623-E7AA24C1906C

Fig. 3

Toxorhina (Ceratocheilus) huanglica Zhang, Li & Yang, 2015 in Zhang et al. 2015: 67. Type locality: China, Yunnan, Lvchun, Huanglianshan, Qimaba.

##### Specimens examined.

China • 1 ♂; Yunnan Province, Mengla County, Xishuangbanna Tropical Rainforest Nature Reserve, Wangtianshu Scenic Spot; 661 m a.s.l.; 21.5090°N, 101.6003°E; 9 July 2016; Qingxia Zhou leg.; light trap; CAU.

##### Diagnosis.

Prescutum and presutural scutum brownish-yellow with three broad dark brown longitudinal stripes. Pleuron dark yellow with two black longitudinal stripes. Wing with cell d closed. R_4_ ending slightly beyond end of basal section of R_5_, m-cu at or slightly before fork of M. Gonocoxite with 1 gonostylus. Gonocoxite dorsally with a blunt, basal lobe covered with numerous setae. Interbase elongated, rod-shaped. Tip of aedeagus bifid with arms short and divergent.

##### Description.

**Figure 3.**
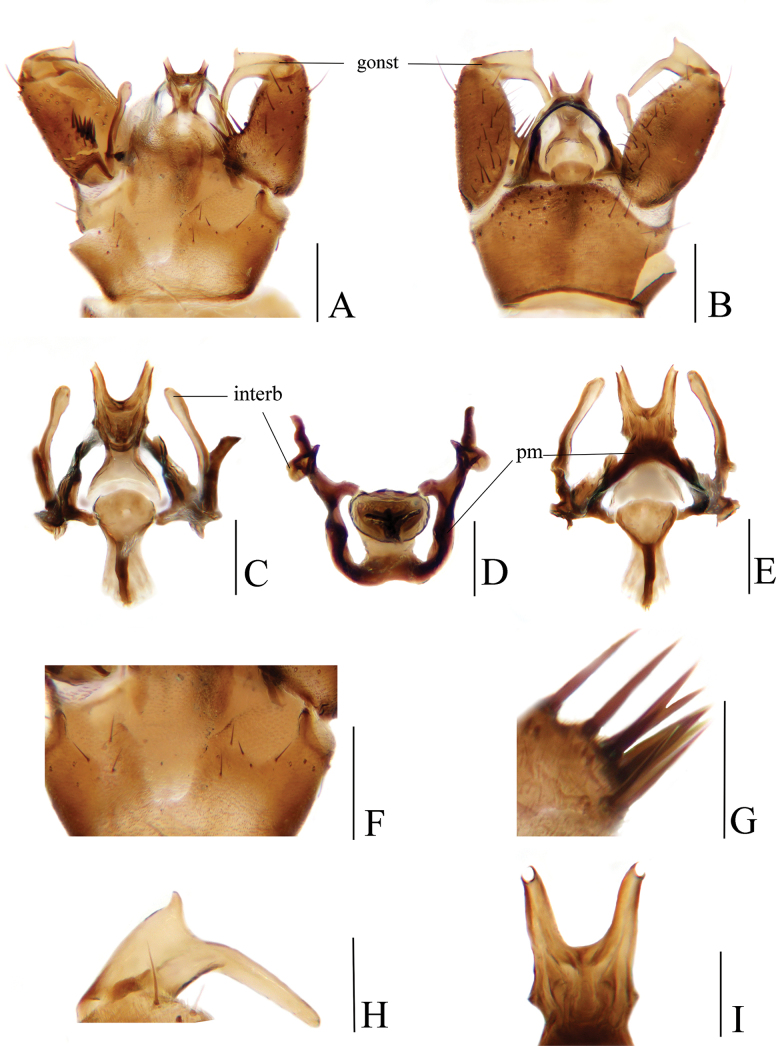
Toxorhina (Ceratocheilus) huanglica**A** male hypopygium, dorsal view **B** male hypopygium, ventral view **C** aedeagal complex, dorsal view **D** aedeagal complex, posterior view **E** aedeagal complex, ventral view **F** posterior margin of tergite 9, dorsal view **G** setae at base of gonocoxite, dorsal view **H** gonostylus, dorsal view **I** tip of aedeagus, ventral view. Scale bars: 0.2 mm (**A, B**); 0.1 mm (**C–F, H**); 0.05 mm (**G, I**).

**Male.** Hypopygium (Fig. 3). Tergite 9 brown with dark brown setae, posterior margin medially with U-shaped deep incision and two translucent areas on both sides (Fig. 3A, F). Gonocoxite dark brown, conical with tip round; dorsal face with a blunt, basal lobe covered with numerous stout setae (Fig. 3A, G); setae on gonocoxite brownish-black (Fig. 3A, B). Gonostylus brown, curved inwards at middle, basal half stout with a longitudinal groove, distal half slender with tip round; outer side of middle with outwardly curved spine (Fig. 3A, B, H). Interbase dark brown, elongated, rod-shaped, about 10 times as long as wide; tip inflated and round (Fig. 3A–C, E). Parameres brownish-black, medially arched fused, distal connecting to base of interbase (Fig. 3C–E). Ejaculatory apodeme brown with middle brownish-black, sheet-like, base narrow (Fig. 3C–E). Aedeagus dark brown, stout at base, tip bifid with arms short and divergent (Fig. 3A–C, E, I).

##### Distribution.

China (Yunnan) (Oosterbroek 2023).

##### Behavior.

The species can be attracted by light.

##### Remarks.

The species was originally described and illustrated by Zhang et al. (2015) based on material collected in China. A more detailed description and illustration of the male hypopygium of this species are provided in this study.

#### ﻿ Toxorhina (Ceratocheilus) pianmica

Taxon classificationAnimaliaDipteraLimoniidae

Xu, Lv & Zhang sp. nov.

4D62C3A0-4333-5E45-BB6C-E25A7BA17E3A

https://zoobank.org/C4EF4120-18B3-44FF-8398-0745D97927D3

Figs 4 , 5

##### Type material.

***Holotype*.** China • ♂; Yunnan Province, Lushui County, Pianma Town; 2123 m a.s.l.; 26.0142°N, 98.6272°E; 6 July 2013; Xuankun Li leg.; light trap; CAU. ***Paratypes***. China • 3 ♂♂; same data as for holotype; CAU.

##### Diagnosis.

**Figure 4.**
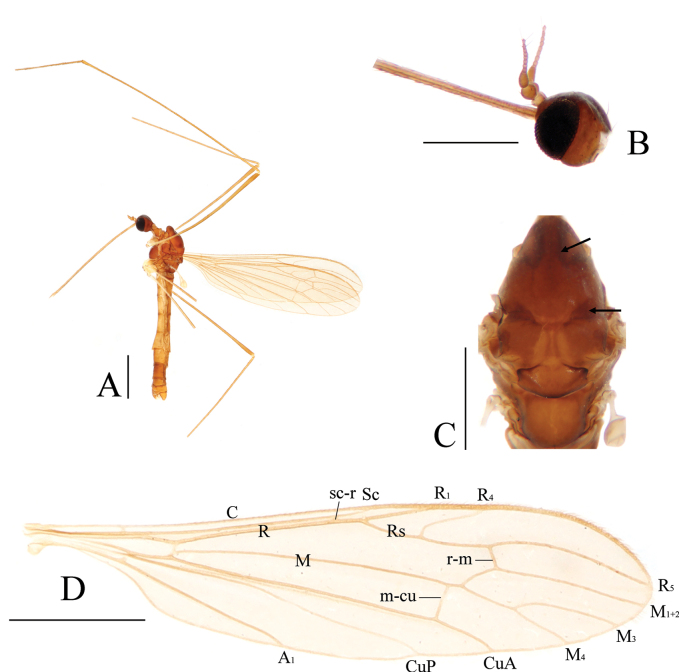
Toxorhina (Ceratocheilus) pianmica sp. nov. **A** habitus of male, lateral view **B** head, lateral view **C** thorax, dorsal view (the arrows refer to the dark areas on prescutum and presutural scutum) **D** wing. Scale bars: 1.0 mm (**A, D**); 0.5 mm (**B, C**).

Prescutum and presutural scutum dark brown with edges of prescutal suture and both sides of caudal edge darker (Fig. 4C). Pleuron dark brown. Wing with cell d open by absence of m-m. R_4_ ending slightly before end of basal section of R_5_, m-cu before fork of M by about 1/3 of its own length. Gonocoxite with 1 gonostylus. Gonocoxite dorsally with three brownish-black, stout preapical setae, and a blunt lobe at inside of base; the lobe with nine stout setae. Interbase elongated, rod-shaped. Tip of aedeagus bifid with arms short and divergent.

##### Description.

**Male** (Fig. 4A). Body length 4.4–4.9 mm (excluding rostrum), wing length 4.5–5.0 mm, rostrum length 2.7–3.0 mm, halter length 0.6–0.7 mm.

Head (Fig. 4B). Dark brown. Setae on head dark brown. Antenna with scape pale brown, pedicel and first flagellomere brown, remaining flagellomeres pale brown. Scape short cylindrical, with dark brown setae. Pedicel globular, with dark brown setae. First flagellomere oval, remaining flagellomeres cylindrical; terminal two flagellomeres longest with pale brown verticils. Rostrum about 3/5 of length of wing, brown with dark brown setae.

Thorax (Fig. 4C). Pronotum brown. Prescutum and presutural scutum dark brown, with edges of prescutal suture and both sides of caudal edge darker. Postsutural scutum dark brown, paler in middle area. Scutellum dark brown with side and caudal edges black, paler in middle area. Mediotergite brown with side edges brownish-black. Pleuron dark brown (Fig. 4A). Coxae yellow with bases brown; trochanters yellow; femora brown with bases paler; tibiae brown; base of fore tarsus brown, remaining tarsi missing. Setae on legs brown. Wing (Fig. 4D) pale brown, without stigma. Veins brown. Venation: Sc ending slightly beyond origin of Rs; sc-r a great distance before tip of Sc; R_4_ ending slightly before end of basal section of R_5_; distal section of R_5_ approach M_1+2_ toward tip; CuP curved suddenly at middle; cell d open by absence of m-m; m-cu before fork of M by about 1/3 its own length. Halter yellow.

Abdomen (Fig. 4A). Segments 1–6 brown with caudal edges darker, segments 7–8 dark brown.

**Figure 5.**
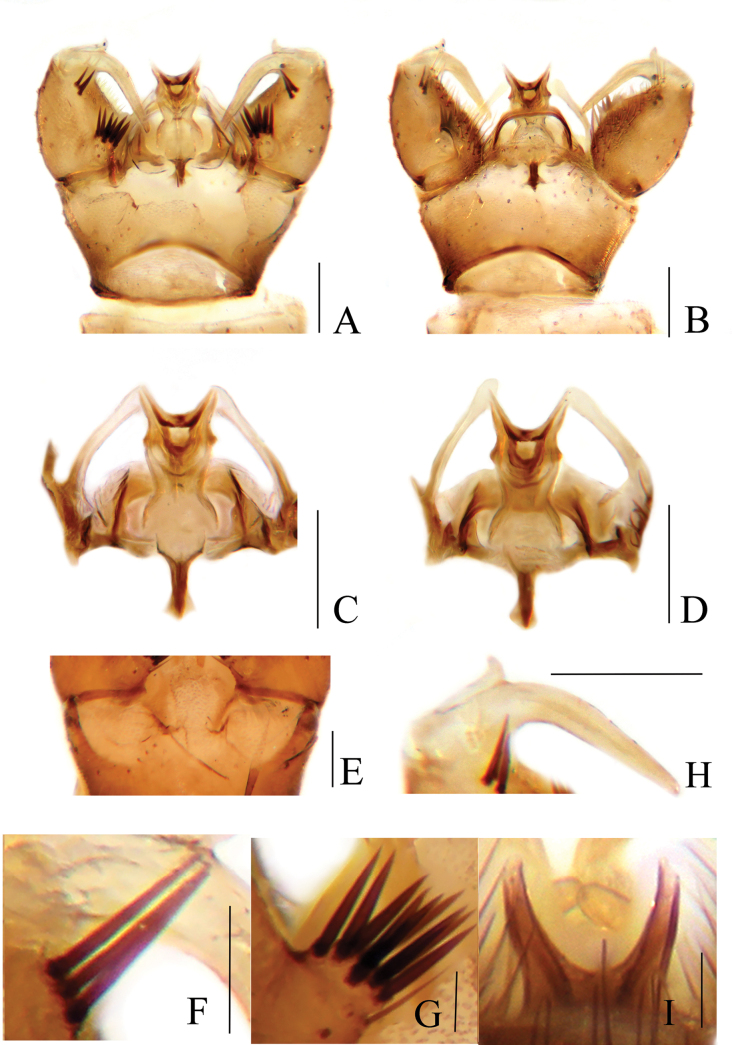
Toxorhina (Ceratocheilus) pianmica sp. nov. **A** male hypopygium, dorsal view **B** male hypopygium, ventral view **C** aedeagal complex, dorsal view **D** aedeagal complex, ventral view **E** posterior margin of tergite 9, dorsal view **F** preapical setae gonocoxite, dorsal view **G** setae at base of gonocoxite, dorsal view **H** gonostylus, dorsal view **I** tip of aedeagus, ventral view. Scale bars: 0.1 mm (**A–E, H**); 0.02 mm (**F, G, I**).

Hypopygium (Fig. 5). Tergite 9 dark brown with dark brown setae, posterior margin with two large translucent areas on both sides (Fig. 5A, E). Gonocoxite brown, conical with tip round (Fig. 5A, B); dorsal face with a few sparse brown setae at outside, three brownish-black, stout preapical setae (Fig. 5A, F), and a blunt lobe at inside of base, this lobe with nine stout setae (Fig. 5A, G); ventral face with numerous brown setae at inside (Fig. 5B). Gonostylus brownish-yellow, curved inwards at basal 1/3; the basal 1/3 stout with a longitudinal groove, distal 2/3 slender with tip round; outer side of basal 1/3 with outwardly curved spine (Fig. 5A, B, H). Interbase pale brownish-yellow, elongated, rod-shaped, about 10 times as long as wide; tip inflated and round (Fig. 5A–D). Parameres dark brown, medially arched fused, distally connecting to base of interbase (Fig. 5C, D). Ejaculatory apodeme dark brown except pale base, rod-shaped (Fig. 5C, D). Aedeagus dark brown, stout at base, tip bifid with arms short and divergent (Fig. 5A–D, I).

**Female.** Unknown.

##### Etymology.

The species is named after the type locality, Pianma Town.

##### Distribution.

China (Yunnan).

##### Behavior.

The species can be attracted by light.

##### Remarks.

The new species is similar to T. (C.) simplicistyla Alexander, 1967 from India in having similar wing venation, but can be separated by the brown abdomen with segments 7–8 darker (Fig. 4A), the gonostylus conspicuously curved with a spine at the basal 1/3 (Fig. 5A, B, H), and the interbase with the tip inflated (Fig. 5A–D). In T. (C.) simplicistyla, the abdomen is brownish-black, the gonostylus is very gently curved with a spine near the middle, and the tip of the interbase is not inflated (Alexander 1967).

#### ﻿ Toxorhina (Ceratocheilus) univirgata

Taxon classificationAnimaliaDipteraLimoniidae

Zhang, Li & Yang, 2015

A6EEEBBC-7CC2-52ED-B381-61A0C6DA58A6

Figs 6 , 7

Toxorhina (Ceratocheilus) univirgata Zhang, Li & Yang, 2015 in Zhang et al. 2015: 76. Type locality: China, Yunnan, Lvchun, Huanglianshan.

##### Specimens examined.

China • 4 ♂♂; Yunnan Province, Lvchun County, Huanglianshan National Nature Reserve, Yakou Protection Station; 1931 m a.s.l.; 22.8956°N, 102.3008°E; 7 July 2016; Qilemoge leg.; light trap; CAU.

##### Diagnosis.

Prescutum and presutural scutum brownish-yellow with three broad brown longitudinal stripes. Pleuron yellow with one dark brown longitudinal stripe. Wing with cell d closed. R_4_ ending beyond end of basal section of R_5_ by 1/3–2/5 of its own length, m-cu from a distance before to at fork of M. Gonocoxite with 1 gonostylus. Gonocoxite dorsally without stout setae. Interbase as a short and flattened plate. Tip of aedeagus bifid with arms very long.

##### Description.

**Figure 6.**
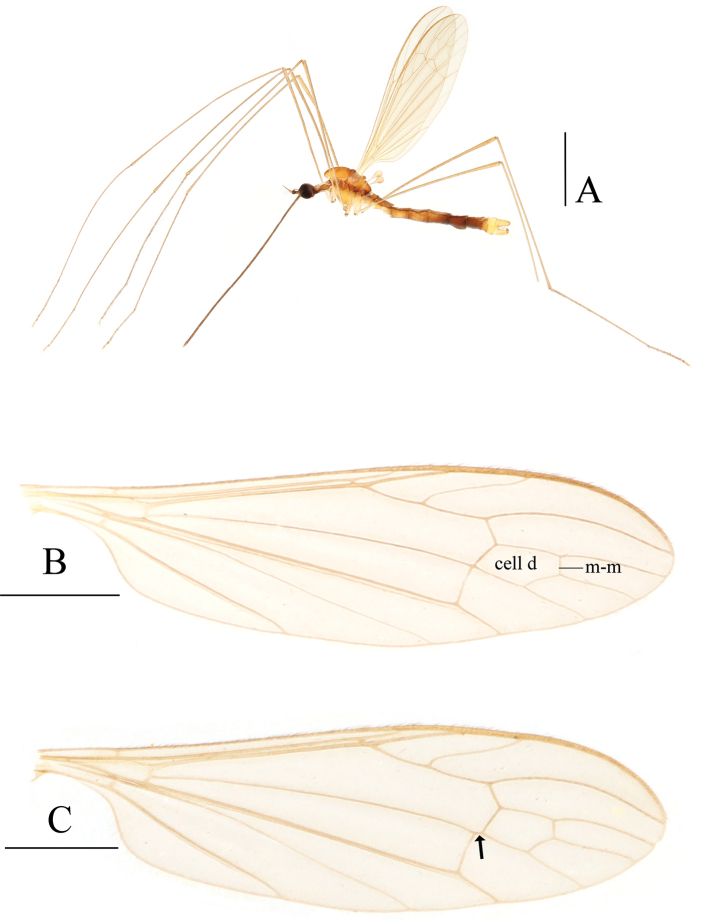
Toxorhina (Ceratocheilus) univirgata**A** habitus of male, lateral view **B** wing **C** variation of wing (the arrow refers to the positional variation of m-cu). Scale bars: 2.0 mm (**A**); 1.0 mm (**B, C**).

**Male** (Fig. 6A). Body length 5.3–5.8 mm (excluding rostrum), wing length 5.0–5.5 mm, rostrum length 4.8–5.3 mm, halter length 0.6–0.7 mm.

Head (Fig. 6A). Rostrum slightly shorter than wing, dark brown with dark brown setae.

Thorax (Fig. 6A). Coxae pale yellow, fore coxa slightly darker; trochanters yellow with tips black; femora brown with bases paler; tibiae brown; tarsi brown with tips slightly paler (Fig. 6A). Wing with position of m-cu unstable, ranging from a distance before fork of M to at fork of M (Fig. 6B, C).

**Figure 7.**
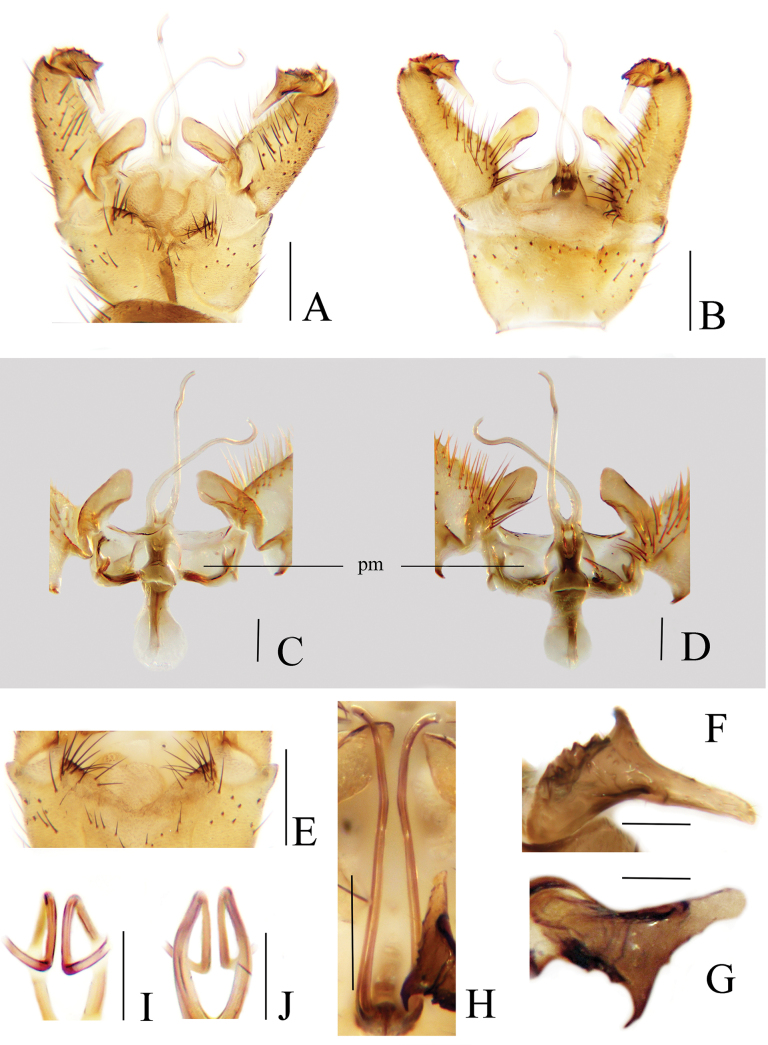
Toxorhina (Ceratocheilus) univirgata**A** male hypopygium, dorsal view **B** male hypopygium, ventral view **C** aedeagal complex, dorsal view **D** aedeagal complex, ventral view **E** posterior margin of tergite 9, dorsal view **F** gonostylus, dorsal view **G** gonostylus, posterior view **H** aedeagus, posterior view **I** tip of aedeagus, dorsal view **J** tip of aedeagus, ventral view. Scale bars: 0.2 mm (**A, B, E**); 0.1 mm (**C, D, H–J**); 0.05 mm (**F, G**).

Hypopygium (Fig. 7). Tergite 9 brownish-yellow with dark brown setae, posterior margin with two nearly triangular lobes, separated by V-shaped incision, laterally with two translucent areas on both sides (Fig. 7A, E). Gonocoxite brownish-yellow, long conical with tip round; setae on gonocoxite dark brown, outside of ventral face without setae (Fig. 7A, B). Gonostylus brown with distal half paler, curved inwards at middle, basal half stout with numerous small spines at outer side, distal half slender with tip round; outer side of middle with a spine; the spine curved outwards (Fig. 7A, B, F, G). Interbase brownish-yellow, as a short and flattened plate, about 3 times as long as wide; tip blunt (Fig. 7A–D). Parameres pale brownish-yellow with edges brown, sheet-like, distally connecting to base of interbase (Fig. 7C, D). Ejaculatory apodeme pale brownish-yellow with middle brown, sheet-like, base narrow (Fig. 7C, D). Aedeagus pale brownish-yellow with brown, stout base; tip bifid, arms filiform and very long, bent dorsally with tip bent outward (Fig. 7A–D, H–J).

##### Distribution.

China (Yunnan) (Oosterbroek 2023).

##### Behavior.

The species can be attracted by light.

##### Remarks.

Zhang et al. (2015) described and illustrated this species as a new species from China. This study supplements the description of the legs, wing, and rostrum. In addition, a more detailed description and illustrations of the male hypopygium of this species are also provided.

## Supplementary Material

XML Treatment for Toxorhina (Ceratocheilus) fuscolimbata

XML Treatment for Toxorhina (Ceratocheilus) huanglica

XML Treatment for Toxorhina (Ceratocheilus) pianmica

XML Treatment for Toxorhina (Ceratocheilus) univirgata

## ﻿Additional information

### Author ORCIDs

Hanhuiying Lv https://orcid.org/0009-0007-2912-5893

Yuanyuan Xu https://orcid.org/0009-0005-2118-0391

Shulin Liu https://orcid.org/0009-0008-6858-6972

Linghui Li https://orcid.org/0009-0003-7432-9482

Kejian Lin https://orcid.org/0000-0002-8531-8506

Xiao Zhang https://orcid.org/0000-0002-8054-0968

### Data availability

All of the data that support the findings of this study are available in the main text.
